# Optimization of Dimensional Accuracy and Surface Roughness of SLA Patterns and SLA-Based IC Components

**DOI:** 10.3390/polym15204038

**Published:** 2023-10-10

**Authors:** Aishabibi Mukhangaliyeva, Damira Dairabayeva, Asma Perveen, Didier Talamona

**Affiliations:** Department of Mechanical and Aerospace Engineering, School of Engineering and Digital Sciences, Nazarbayev University, Astana 010000, Kazakhstan; aishabibi.mukhangaliyeva@alumni.nu.edu.kz (A.M.); damira.dairabayeva@alumni.nu.edu.kz (D.D.); asma.perveen@nu.edu.kz (A.P.)

**Keywords:** rapid investment casting, stereolithography, dimensional accuracy, surface roughness, Taguchi, optimization, printing parameters

## Abstract

Rapid investment casting is a casting process in which the sacrificial patterns are fabricated using additive manufacturing techniques, making the creation of advanced designs possible. One of the popular 3D printing methods applied in rapid investment casting is stereolithography because of its high dimensional precision and surface quality. Printing parameters of the used additive manufacturing method can influence the surface quality and accuracy of the rapid investment cast geometries. Hence, this study aims to investigate the effect of stereolithography printing parameters on the dimensional accuracy and surface roughness of printed patterns and investment cast parts. Castable wax material was used to print the sacrificial patterns for casting. A small-scale prosthetic biomedical implant for total hip replacement was selected to be the benchmark model due to its practical significance. The main results indicate that the most significant stereolithography printing parameter affecting surface roughness is build angle, followed by layer thickness. The optimum parameters that minimize the surface roughness are 0.025 mm layer thickness, 0° build angle, 1.0 support density index, and across the front base orientation. As for the dimensional accuracy, the optimum stereolithography parameters are 0.025 mm layer thickness, 30° build angle, 0.6 support density index, and diagonal to the front base orientation. The optimal printing parameters to obtain superior dimensional accuracy of the cast parts are 0.05 mm layer thickness, 45° build angle, 0.8 support density index, and diagonal to the front model base orientation. With respect to the surface roughness, lower values were obtained at 0.025 mm layer thickness, 0° build angle, 1.0 support density index, and parallel to the front base orientation.

## 1. Introduction

Investment casting (IC), also referred to as lost wax casting or precision casting, is a near-net shape process of manufacturing complex metal geometries. IC can be used to cast non-ferrous and ferrous metals and produces parts with high dimensional accuracy and excellent surface finish. IC is widely used in numerous industries including automotive, aerospace, medicine, military, jewelry, and energy sectors [1].

**Figure 1:**
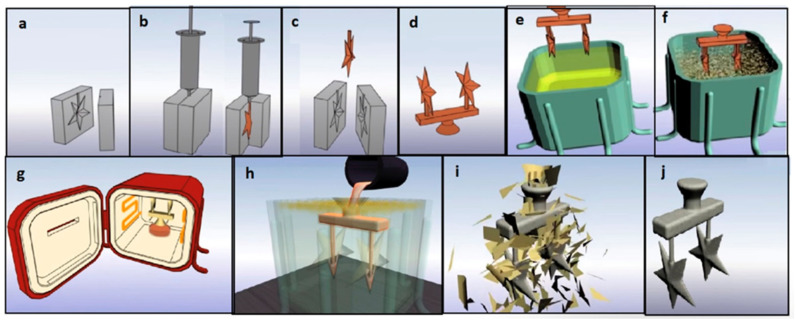
Conventional investment casting: (**a**) Preparing a metal mold for wax injection; (**b**) injecting wax to create patterns; (**c**) ejecting wax from the mold; (**d**) assembling wax patterns into a tree; (**e**) ceramic slurry coating; (**f**) coating with stucco; (**g**) dewaxing by heating; (**h**) pouring metal; (**i**) removing a ceramic shell; (**j**) a cast part that is prepared to be severed from the assembly [1].

The conventional IC process is shown in Figure 1. First, a metal mold for a wax pattern is manufactured through the fabrication of a metal die. The molten wax is then injected into the metal mold to create a wax pattern. A casting tree is assembled with multiple wax patterns. Afterward, the casting tree is dipped into a ceramic slurry containing alumina and colloidal silica to create the interior walls of a ceramic mold. The obtained mold is heated in an oven to melt all wax content. The molten metal is poured into the ceramic mold and allowed to solidify and cool before the destruction of the ceramic shell [2].

One of the issues faced with conventional IC is the manufacturing of wax patterns since it requires a difficult tooling process. The pattern-making stage can be simplified by using additive manufacturing (AM) to replace the IC steps such as the hard tooling of metal molds and wax injection. AM, also known as rapid prototyping, is a layer-by-layer fabrication process that emerged in the 1980s. In recent decades, AM technologies evolved significantly to be used in IC. The AM-assisted IC is called rapid investment casting (RIC) due to the reduced time needed to prepare the patterns for casting [3].

The AM technologies in RIC are used either through an indirect or direct approach. A 3D-printed mold is used to prepare the wax pattern in the indirect RIC, while the pattern is fabricated using a printer in the direct RIC [4]. The application of AM-based sacrificial patterns in IC reduces the lead time and cost significantly for individual and small-size productions when compared to the conventional process. The AM processes used for RIC include stereolithography (SLA), fused filament fabrication (FFF), selective laser sintering (SLS), and laminated object manufacturing (LOM). In order to create castings of superior quality, the AM-based patterns are required to have a low melting point, sufficient mechanical strength and toughness, low surface roughness, high dimensional accuracy, and no residual ash following burnout [5,6]. SLA is regarded as a very precise method among 3D printing techniques [7].

**Figure 2:**
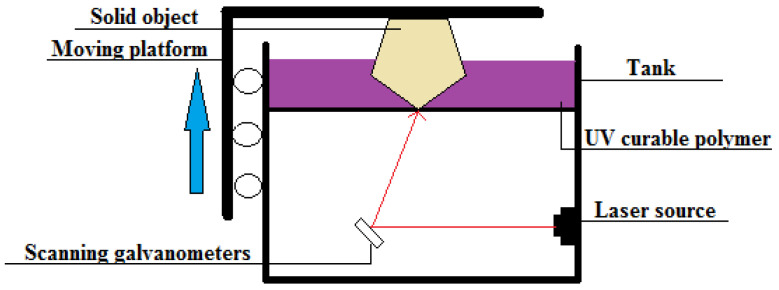
A general schematic of the SLA process (bottom-up).

SLA is a vat photopolymerization technology that uses ultraviolet (UV) light to cure or solidify liquid resin, a photosensitive thermoset material. The UV radiation initiates the curing reaction of a resin which results in the cross-linking of molecular chains and the polymerization of oligomers and monomers. Depending on the printing device, there might be a bottom-up or top-down method. Indeed, desktop 3D printers, such as Formlabs 3D printers, employ the bottom-up approach, whereas industrial 3D printers use the top-down technique [8]. As shown in Figure 2, the main components of the SLA machines are the laser, mirrors, galvanometers, resin tank, and build platform. A layer of liquid photopolymer is scanned by a laser to harden the resin in the areas specified by the CAD model. After the curing of one layer, the surface is covered with a thin film of resin for further exposure to the laser beam. The platform is raised/moved up along the *z*-axis for the curing of each consecutive layer [1].

There are several benefits of casting with SLA. Firstly, compared to traditional IC, its manufacturing cost and lead time are decreased. Secondly, waste is reduced as a result of the faster machining [9,10]. Also, it is possible to implement modifications to the model when designing without affecting the production time [1].

Most of the SLA studies considered the effect of the layer thickness, position on the build platform, and build angle on surface roughness or dimensional accuracy of SLA parts. A summary of the literature review is given in Table 1.

Khaledi et al. and Piangsuk et al. have studied the dimensional accuracy of SLA-based RIC and milled copings used in prosthetic dentistry [22,23]. According to the outcomes, both SLA-printed and SLA-based RIC components demonstrated higher dimensional accuracy than milled components.

**Figure 3:**
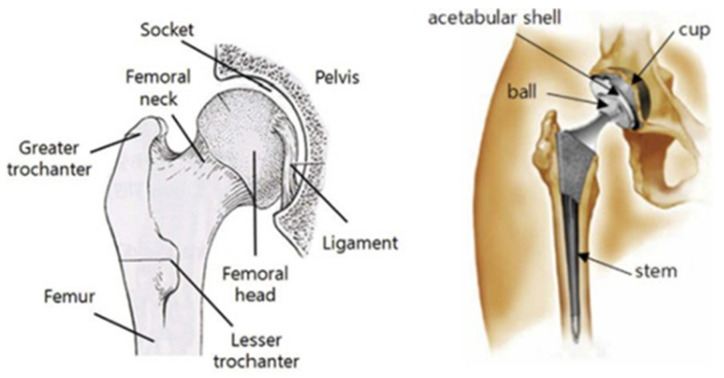
Parts of a hip implant [24].

A prosthetic biomedical implant for total hip replacement was selected to be the benchmark model due to its practical significance based on the available literature. A hip implant is used to replace a hip joint, one of the largest ball-and-socket joints in the human body, which allows for the free movement of a leg, supports the upper part of the body, and absorbs the movement impact. As shown in Figure 3, the hip implant consists of three main parts, such as the cup, ball, and stem. The cup is a replacement for the hip socket in the pelvis bone. The ball placed in the cup is inserted into the acetabular shell, while the stem is put into the femoral canal for stability [24].

Several researchers have studied the dimensional accuracy and surface roughness of hip implants produced by FFF and FFF-based IC. A study by Singh et al. investigated the effect of IC process parameters such as slurry viscosity, number of slurry layers, and dry time of the primary coating on the surface roughness of hip joints developed by a combination of IC and FFF [25]. The design was used the femoral ball and shortened stem. It was found that the most influential factor on the quality of a cast hip implant surface is the number of layers. Kumar et al. examined three hip implants with different volume-to-surface (V/A) ratios [26]. The first model consisted of only a stem, while the other two implants were designed with a femoral ball. Researchers focused on both the printing and casting parameters such as the orientation, density of the pattern, mold thickness, grade of material, type of pattern, and V/A ratio. According to the results, an orientation of 0° angle is recommended for Acrylonitrile Butadiene Styrene (ABS) patterns while a wax layer notably increased the surface quality of cast parts. A simplified model of the hip implant was designed by Tiwary et al., and the printing parameters examined in this article were extrusion speed, infill, number of top and bottom solid layers, and layer thickness [27]. It was found that the surface roughness of the FFF parts can be decreased by making the top and bottom layers solid and it was suggested that the RIC technique be applied for a low volume of production.

In the RIC, the process parameters used to print the sacrificial patterns can affect the surface roughness and dimensional accuracy of the castings. Hence, the surface finish and accuracy of the printed patterns must be improved by optimizing the printing parameters. According to the literature review, numerous studies focused on the dimensional accuracy and surface roughness of the SLA-printed parts separately. However, there is lack of studies on the effects of the SLA process parameters on the surface roughness and dimensional accuracy of both printed and cast parts and on castable wax resin material. Consequently, this study intends to examine and fill these research gaps. The aim of this work is to investigate the effect of the SLA printing parameters on the dimensional accuracy and surface roughness of the printed patterns and the cast components. The following objectives were defined to attain the aim of this study:

- Design a benchmark model of the small-scale prosthetic biomedical hip implant.
- Fabricate benchmark patterns using the SLA printing technology and assess their dimensional accuracy and surface roughness.
- Cast and measure the dimensional accuracy and surface roughness of the AM-based IC components.
- Examine the experimental results and obtain the optimized printing parameters for dimensional accuracy and surface roughness.
- Investigate the hardness, microstructure, and composition of the IC parts.

## 2. Materials and Methods

This section describes the experimental procedure, model design, printing materials, design of experiments, and equipment used in the study.

### 2.1. Experimental Procedure

**Figure 4:**
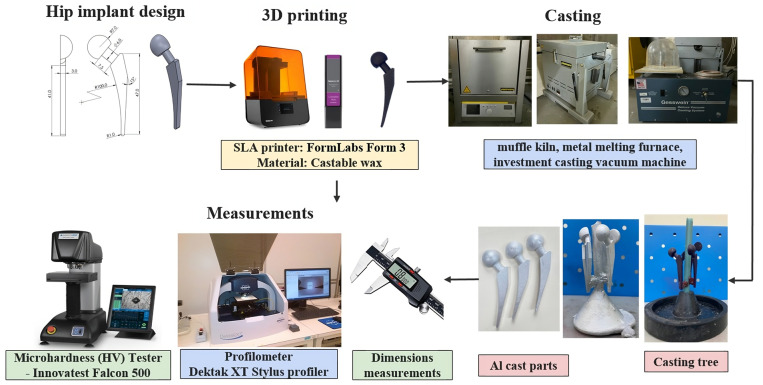
Experimental procedure.

This study consists of several stages as shown in Figure 4. Firstly, the virtual model of the hip implant was created using a computer-aided design software, SolidWorks 2021. The material and printing parameters were selected. Taguchi’s L18 orthogonal array was chosen for the design of experiments (DOE). The SLA printer, FormLabs Form 3 (Formlabs Inc., Somerville, MA, USA), was used to print the specimens. The surface roughness and dimensional accuracy of the printed parts were measured and processed to examine the effect of parameters on these properties. Afterward, the printed samples were used to fabricate the mold for the casting. The IC parts were produced using aluminum. The surface finish and dimensional deviation of these parts were measured. The microhardness and microstructure of the IC parts were characterized. 

### 2.2. Benchmark model design

**Figure 5:**
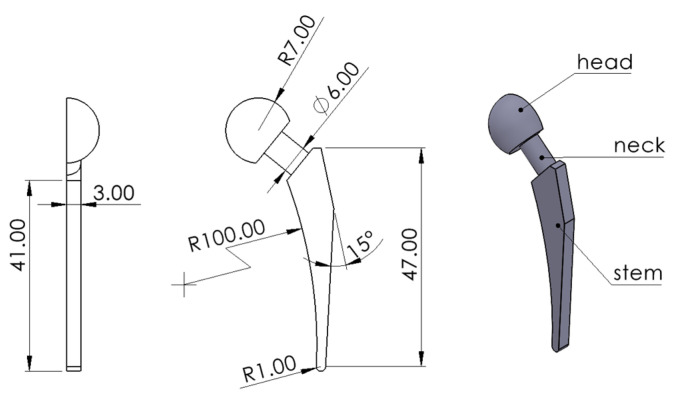
Geometry and feature labeling of the hip implant model.

The small-scale prosthetic biomedical hip implant model designed for the experiment is shown in Figure 5. The model was created using the SolidWorks 2021 software based on the literature review and resized down to an appropriate scale for 3D printing [22,23,24]. The main geometric features of the hip implant are the femoral head, the neck, and the stem. The model was designed to be the half of the implant since the part was required to lie flat on the surface for measurement purposes.

### 2.3. SLA Printing of Patterns

The SLA patterns were printed using FormLabs Form 3. The printer was equipped with 250 mW laser power and low-force stereolithography (LFS) technology. The size of the laser spot was 85 µm [28].

The material used for the SLA printing of patterns was FormLabs Castable Wax V1 (FLCWPU01) resin, an acrylate photopolymer with a wax content of 20%. This resin produced the pattern for casting with a high level of detail, with print resolutions of 25 and 50 µm and a superior surface finish. The castable wax resulted in a clean burnout and almost zero ash content. The layer thicknesses that can be printed with this material are 0.025 and 0.05 mm. According to the material specifications, no-post curing is needed for the castable wax [29]. The characteristics of the resin are given in Table 2. The Preform 3.27.1 software was used to set up the parts for SLA printing.

### 2.4. Design of Experiment

**Figure 6:**
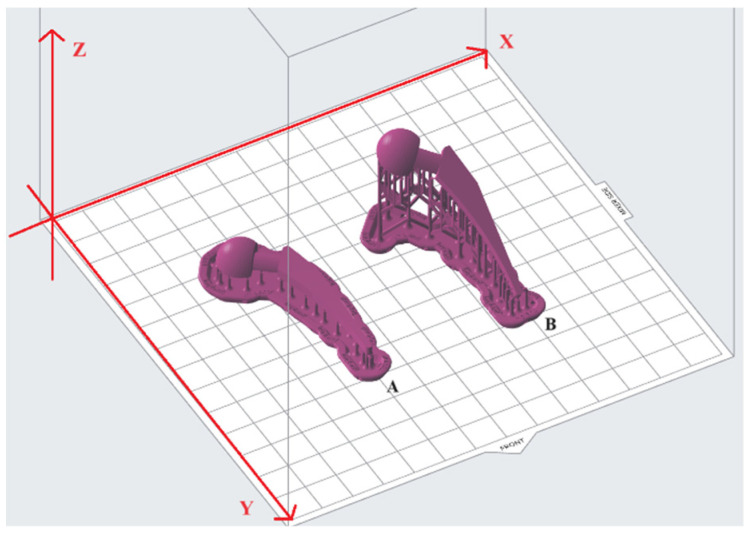
Build angle of 0° (**A**) and 30° (**B**), across the front model base orientation.

**Figure 7:**
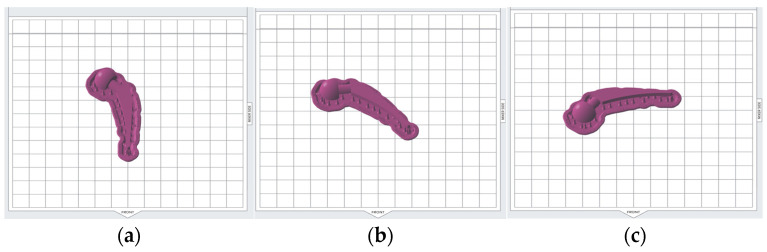
Model base orientations (build angle of 0°); (**a**) across the front; (**b**) diagonal to the front; (**c**) parallel to the front.

The Taguchi method of parametric design of experiment (DOE) was employed to examine the impact of the selected printing parameters (see Table 3) on the dimensional accuracy and surface roughness of the hip implant model. The Taguchi method allows for a reduction in the number of experiments while still determining the sensitivity and importance of each parameter. In this study, the process parameters of SLA printing were chosen to be layer thickness, build angle (see Figure 6), support density, and model base orientation (see Figure 7). In Figure 6, model base orientations are across the front; build angles are 0° and 30° in part A and B, respectively. To obtain B, the A was rotated along the *x*-axis by 30°. Then, the two other cases where the build angle was 30° and model base orientation was diagonal to the front and parallel to the front were obtained by rotating B along the *z*-axis by 45° and 90°, respectively. The support density was chosen in the range [0.6, 1.0] because a support density of less than 0.6 was not recommended and showed an error by the Preform software when the checking model for printability while using a support density of more than 1.0; surface finish was considerably reduced due to the footprints of the excess support structures. According to the chosen DOE, there should be 3 levels of support density, therefore, 0.6, 0.8, and 1.0 were chosen.

**Figure 8:**
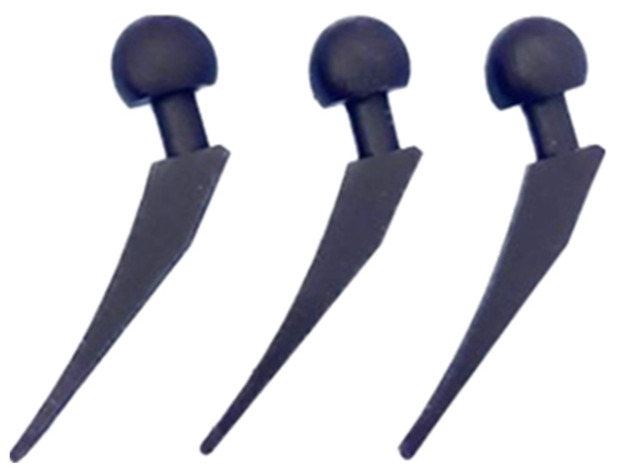
Hip implant printed using SLA.

The Minitab Statistical Software Version 21.1.0 was used to create Taguchi’s experimental design. L18 Orthogonal Array with 18 rows as shown in Table 4 was created for the chosen factors and levels. There is a setting for the different experimental SLA printing runs in each row. Each experimental run was repeated three times as shown in Figure 8.

### 2.5. Investment Casting

**Figure 9:**
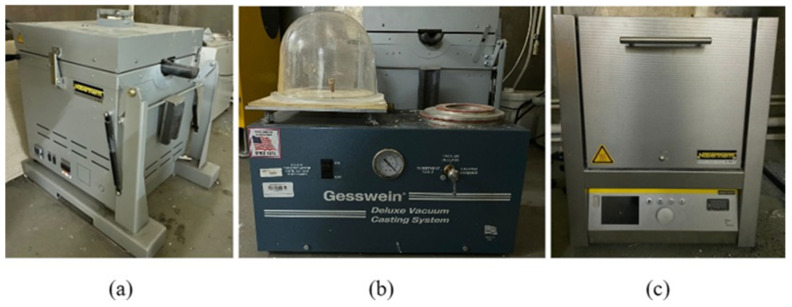
Equipment used for casting (**a**) metal melting furnace; (**b**) IC vacuum machine; (**c**) muffle kiln.

Aluminum wrought alloy (AMg3 (1530), Kazakhstan Metal Industrial Company, Astana, Kazakhstan) was chosen as the casting metal for this experiment since it is a lightweight, highly recyclable, inexpensive, and available metal with good strength, stiffness, and high thermal conductivity. The equipment used for casting procedures is shown in Figure 9.

**Figure 10:**
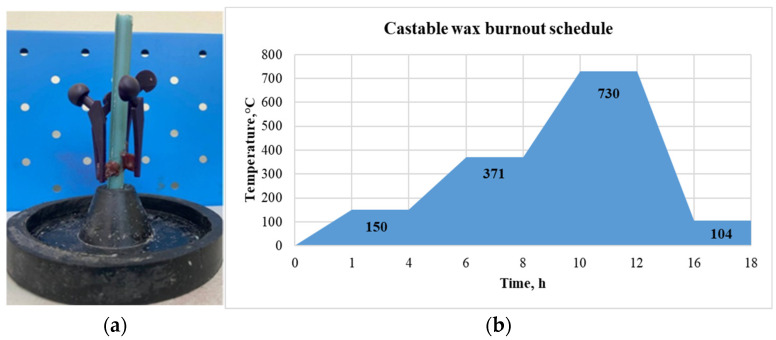
(**a**) Casting tree; (**b**) burnout cycle of the castable wax.

Firstly, wax rods were used to build the patterns in a casting tree, as can be seen in Figure 10a. Then, the mold creation was carried out using Ultravest investment powder from Ranson and Randolf and distilled water in a 39/100 water to powder ratio. According to the instructions for the investment powder, 690 g of powder and 269.4 g of water were mixed for an investment in a flask with a 10.2 cm height and 8.3 cm diameter. After mixing the powder with water for 2–3 min, the investment table side of the Gesswein Deluxe Vacuum Casting System machine was utilized to remove trapped air from the mixture. The mixture was then added to the flask containing the casting tree, and the vacuum was applied once again. Mold reaches its peak green intensity after 2 h, thus it was left to sit undisturbed for that time period.

For further burnout of the flask with investment, Nabertherm Muffle Furnace L15/11/B410 (Nabertherm GmbH, Lilienthal, Germany) was used. The flask was put into a kiln which was preheated to 150 °C. The burnout cycle for the selected castable wax material was approximately 15 h as illustrated in Figure 10b. Aluminum bars were placed into a Nabertherm K1/13 (Nabertherm GmbH, Lilienthal, Germany) metal melting furnace and melted at 750 °C during the final two hours of the burnout cycle. The casting parameters used were as follows: casting temperature of 750 °C, mold temperature before casting of 104 °C, pouring speed of 2.5 cm/s, and time before cooling 20 min.

**Figure 11:**
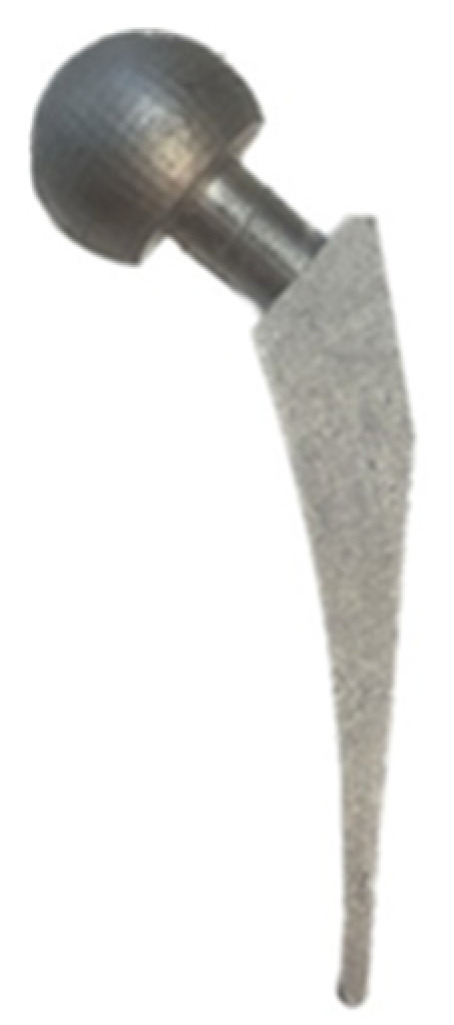
Hip implant produced using IC.

The flask was taken out of the kiln and put into the casting chamber of the Gesswein Deluxe Vacuum Casting System machine (Gesswein, Bridgeport, CT, USA) after 1.5 h of gradually melting the metal. The vacuum was switched on and the molten aluminum metal was poured into the flask. After 15–20 min of metal solidification, the hot flask was cooled with water and the investment casting shells were removed from th e cast parts. Next, the cast hip implant parts were sawed off from the casting tree and no post-processing was carried out. The hip implants produced using IC are shown in Figure 11.

### 2.6. Measurements

A Vernier digital caliper was used to measure the dimensional accuracy of the printed components. The features that were measured and checked for accuracy are shown in Table 5 and Figure 5.

The surface roughness was measured using a profilometer—Dektak XT Stylus (Bruker, Billerica, MA, USA) profiler. The profilometer was in contact with the sample using a stylus with a single-crystal diamond tip with a radius of 12.5 µm. The sample stage slid the sample underneath the stylus tip to draw a profile while the equipment maintained a steady stylus force. This device assessed the surface roughness by profiling the surface topography. The surface roughness was measured in the stem of the implant. The arithmetical average roughness (*Ra*) refers to the arithmetic mean of all the absolute variances from the center line over the total length and is defined by Equation (1).

(1) $$R_{a}=\frac{1}{L}\;\int_{0}^{L}\left|z\left(x\right)\right|dx\;$$

where *L* is a sampling length and *z* is a coordinate of the curve profile [30].

The measurement setup parameters for the profilometer were the following: duration of 20 sec, length of 5000 µm, range of 524 µm, and 7 mg of stylus force. An average of three measurements was taken for each specimen.

The Vickers microhardness of the SLA-based IC was measured using the Microhardness (HV) Tester—Innovatest Falcon 500 (INNOVATEST Europe BV, Maastricht, The Netherlands). The hardness was measured three times for each specimen. A load of 0.5 kg was applied to the specimen with a dwelling time of 10 s. The surface of the parts was polished using the polishing machine before the test.

The microstructure was examined using the Scanning Electron Microscope JEOL JSM-IT200 (JEOL Ltd., Tokyo, Japan). The Energy Dispersive Spectroscopy (EDS) analysis was conducted to investigate the chemical composition of the specimen while imaging. The hip implants were cut using a micro wire electrical discharge machine (µWEDM) to prepare the samples for the SEM. The surface of the samples was polished using a polishing machine. The samples also underwent wet etching to remove the material from the surface.

## 3. Results and Discussion

This section represents the results of the experiments and analysis.

### 3.1. Analysis of Measurements

The S/N ratio for the smaller-is-better category was selected to minimize the surface roughness and dimensional deviation of the printed patterns. The S/N ratio is calculated using Equation (2).

(2) $$\eta =-10log(\frac{1}{n}\sum_{i=1}^{n}\gamma_{i}^{2})$$

where *η* is an S/N ratio, *γ_i_* is a response for a given factor level combination, and *n* is a number of responses [30].

### 3.2. SLA Printed Patterns: Dimensional Accuracy

The dimensions of all the printed components were recorded and compared with the nominal values. The average deviations of all features are demonstrated in Table 6. Both expansion and shrinkage occurred since the average deviation from the nominal values varied between −0.21 mm and 0.12 mm. The lowest deviation corresponded to the neck length, while the stem length had the highest average deviation. Both the stem and neck thicknesses of the printed parts were larger than the nominal dimensions.

The mean and S/N rankings of the parameters for the dimensional accuracy of each feature are given in Table A1 in Appendix A, where 1 and 4 correspond to the most and least significant printing parameter, respectively. The sum of the rankings of each parameter was calculated and, based on this, the significance of factors was revealed. Both the mean and S/N rankings gave the same outcomes. Based on their influence on the dimensional accuracy of the SLA printed parts, the most significant parameter was the build angle, followed by the support density, model base orientation, and layer thickness.

The optimal level for each parameter was determined for each feature measurement using the mean analysis. The results are demonstrated in Table A2 in Appendix A. To obtain the superior dimensional accuracy of the printed components, the number of occurring frequencies of each level for each factor was added, and the level with the highest frequency was selected as the optimal level.

### 3.3. SLA Printed Patterns: Surface Roughness

The roughness of all SLA samples was measured and the average surface roughness, standard deviation, and S/N ratio value for each experimental run are given in Table 7. The highest surface roughness (2.927 µm) was measured for Run 14, while the lowest value (0.882 µm) was recorded for Run 2.

**Figure 12:**
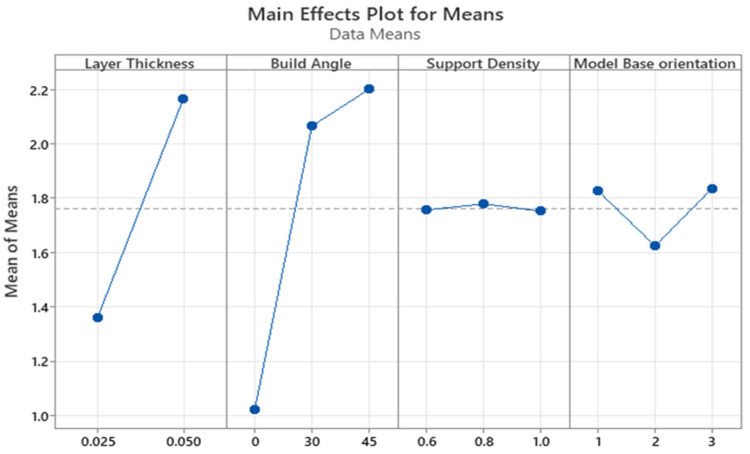
Main effects plot for the means for surface roughness of SLA printed parts.

The main effects plot and response table for the means are shown in Figure 12 and Table 8, respectively. The most influential process parameter on the surface roughness was the build angle with a contribution of 59.11%. The dependence of the surface roughness on the build angle is monotonic since surface roughness is minimized with the decreasing build angle. The lowest surface roughness was recorded for the 0° angle, which is in line with the available findings. This can be explained by the staircase phenomenon, which is intrinsic to the layer-by-layer printing processes and more vivid on sloped surfaces [31,32].

The second most significant printing parameter was the layer thickness. The contribution of this parameter was 24.66%. The correlation between the surface roughness and layer thickness was also monotonic as the roughness decreased with a lower layer thickness. This finding agrees with the current literature [33,34]. Hence, the staircase effect can be minimized by selecting the appropriate values of build angle and layer thickness.

The model base orientation and support density showed the lowest effect on the surface roughness of the printed parts, with a contribution of 1.28% and 0.10%, respectively. The support density of 1.0 and across the front base orientation resulted in a slightly lower surface roughness. The *p*-values also indicate whether a particular process parameter significantly affects surface roughness. As given in Table 9, the *p*-values of the build angle and layer thickness were 0.0003 and 0.0022, respectively. Since both *p*-values are less than the significance level of 0.05, the association between these process parameters and surface roughness is statistically significant. On the contrary, the effect of support density and model base orientation was statistically insignificant. The *p*-values of support density (0.9645) and model base orientation (0.6613) were greater than the significance level.

The optimized printing parameters for the dimensional accuracy and the surface roughness are given in Table A3 in Appendix A. For the dimensional accuracy, the optimum printing parameters are a layer thickness of 0.025 mm, build angle of 30°, 0.6 support density, and diagonal to the front base orientation. To minimize the surface roughness, a 0.025 mm layer thickness, 0° build angle, 1.0 support density, and across the front base orientation are recommended to be used.

For validation, the SLA parts were printed with the optimized parameters, and their dimensional accuracy and surface roughness were measured. They gave better results than the SLA parts printed with the non-optimized parameters. The average surface roughness was equal to 0.906 µm. From the outcomes of dimensional accuracy measurements shown in Table A4 in Appendix A, it can be noted that average dimensional deviations of the SLA parts printed with the optimized parameters ranges from −0.06 to 0.15 mm.

### 3.4. SLA-Based IC Parts: Dimensional Accuracy

The dimensions of all SLA-based IC parts were recorded and compared with the nominal values, with the average deviations of all features given in Table 10. The average deviation from the nominal values ranged between −0.40 mm and 0.15 mm. Similar to the SLA printed patterns, the lowest deviation of the cast parts from the nominal value corresponded to the neck length, whereas the stem length had the highest average deviation. The sphere radius and neck and stem thicknesses were larger than the nominal dimensions.

**Figure 13:**
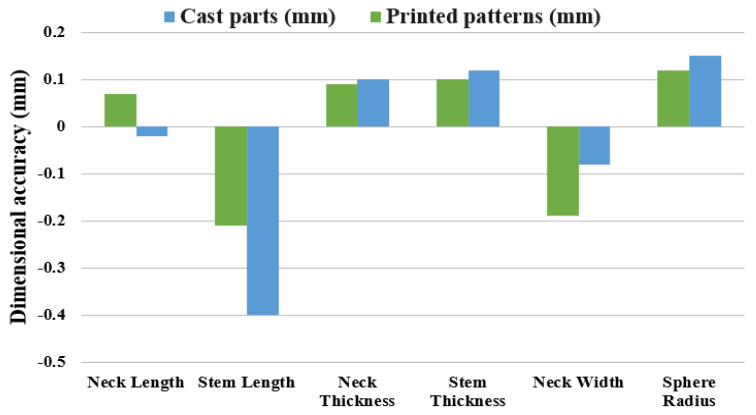
Comparison between the SLA patterns and the cast parts.

The comparison between the SLA patterns and the cast parts in terms of the dimensional deviation from the nominal value is shown in Figure 13. Moreover, it is important to note that, when calculating dimensional deviation, the average measured values of both the SLA printed and the SLA-based IC parts were compared with the nominal value from the CAD file. The features such as neck thickness, stem thickness, and sphere radius had higher values of positive deviation (oversized) in the cast parts compared to the printed patterns. In the case of the neck width, the printed patterns had a larger negative deviation (undersized), which means that the expansion of the cast parts was present. This trend was present for the majority of the features. In contrast, the shrinkage of the cast parts was observed in the two length features. The stem length saw further shrinkage and had a negative deviation in both the printed and the cast parts. As for the neck length, the printed patterns were oversized, while the cast parts were undersized. This is possibly due to the shrinkage of the wax patterns during the burnout and the shrinkage of the aluminum during the metal solidification in the Z-direction as the patterns were attached to the feed sprue vertically [35].

The common assumption in IC (vacuum-assisted utilizing plaster molds) is that the casting samples will be slightly smaller than the original designs due to the phenomena of solidification shrinkage. However, there can be cases in which the casting samples are bigger than the patterns, and such occurrences can be related to several factors. When castable wax patterns are melted to form the investment mold, they may initially expand significantly and therefore the IC parts can have dimensions that are bigger than those of the original patterns [35]. Furthermore, larger castings might arise from non-uniform shrinkage caused by variations in the cooling rates of mold and metal, and particularly from the thicker to thinner areas of the part [36]. Piwonka [37] stated that the mold’s deformation needs to be taken into consideration to estimate the final dimensions of the IC parts. The heating and cooling processes, including the preheating, burnout, and dewaxing, and pouring can alter the casting dimensions of the mold [38].

The mean ranking of the parameters for the dimensional accuracy of each feature is given in Table A5 in Appendix A. Based on their influence on the dimensional accuracy of the cast parts, the most significant parameter was the build angle, followed by the layer thickness. The support density and the model base orientation were the least important factors.

The optimal level for each parameter was determined for each feature measurement using the mean analysis. As given in Table A6 in Appendix A, the optimal printing parameters to obtain superior dimensional accuracy in the cast parts are a 0.050 mm layer thickness, 45° build angle, 0.8 support density, and diagonal to the front model base orientation.

### 3.5. SLA-Based IC Parts: Surface Roughness

The average surface roughness results of the SLA-based IC parts, standard deviation, and S/N ratio value for each experimental run are given in Table 11. The highest surface roughness (3.139 µm) was measured for Run 7, which was printed with a 0.025 mm layer thickness, 45° build angle, 0.6 support density, and across the front model base orientation. In contrast, the lowest value (1.406 µm) was recorded for Run 1 as shown in Table 3 and Table 4, with printing parameters such as a 0.025 mm layer thickness, 0° build angle, 0.6 support density, and parallel the front model base orientation.

**Figure 14:**
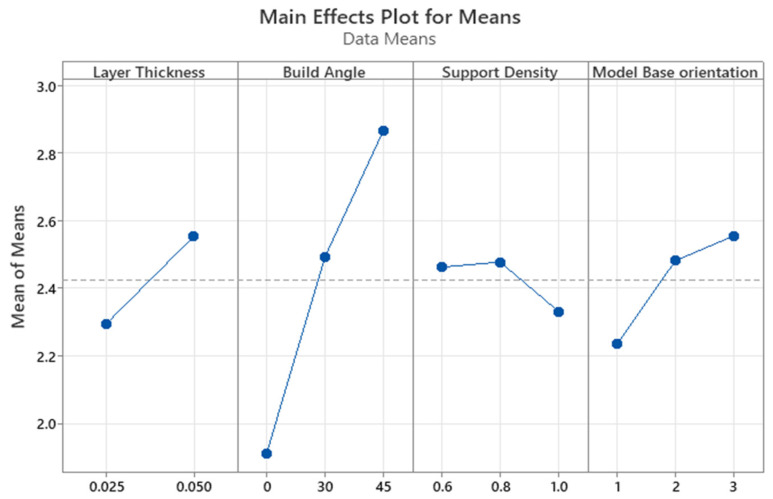
Main effects plot for means for surface roughness of SLA-based IC parts.

The main effects plot and the response table for means are illustrated in Figure 14 and Table 12, respectively. The build angle and the layer thickness were revealed to be the parameters with the greatest impact. The 0° angle had the lowest surface roughness, which is consistent with the data provided by Federov et al. [39]. The staircase effect, which is inherent to layer-by-layer printing methods and is more pronounced on sloping surfaces [1], can be utilized for clarifying phenomena. Layer thickness was the second most important printing parameter. Surface roughness and layer thickness have a monotonic relationship, with roughness decreasing as layer thickness decreases. The result is consistent with the findings of Jacobs [40]. Therefore, by choosing proper values for the build angle and the layer thickness, the staircase effect may be reduced.

The model base orientation and the support density showed statistically negligible effects on surface roughness according to the ranking of process parameters. The surface roughness was lowered with increasing the support density and with changing the model base orientation from diagonal to the front to parallel to the front. Lower roughness values were obtained with 1.0 support density.

The build angle was the process parameter that had the greatest impact on the surface roughness, as shown in Table 13, with a contribution of 48.98%. The layer thickness, which contributed 6.86% of the total variance, was the second most important component. The model base orientation and support density each made 4.75% and 1.51% of the total contribution.

Additionally, the *p*-values show whether a certain process variable substantially influences the surface roughness. The build angle has a *p*-value of 0.016. The correlation between this process parameter and surface roughness is statistically significant since the *p*-value is under the threshold of 0.05 for significance. On the other hand, due to the exceeded significance threshold, the effects of the layer thickness (0.208), support density (0.822), and model base orientation (0.554) were insignificant according to the statistical analysis.

Furthermore, it can be noted that the percentage contribution of residual error for the surface roughness of the SLA-based IC components is higher (37.90%) compared to the percentage contribution of the other sources such as layer thickness, build angle, support density, and model base orientation (see Table 13). The residual error is known as the amount of data variance left unaccounted for by the model in an ANOVA table. A large percentage contribution of residual error suggests that there could be other factors or variables impacting the response variable but not being taken into account by the model. Consequently, more research may be required to find and resolve any potential causes of bias or mistakes in the model.

The optimized printing parameters for dimensional accuracy and surface roughness of the SLA-based IC components are presented in Table A7 in Appendix A. To achieve better surface roughness results, a layer thickness of 0.025 mm, build angle of 0°, support density of 1, and parallel to the front model base orientation should be applied.

For the validation of the results, the SLA parts were printed with optimized parameters for the SLA-based IC components, and using those patterns the casting was performed. The average surface roughness of the SLA-based IC parts with the optimized printing parameters was equal to 1.462 µm. The results of dimensional accuracy measurements are demonstrated in Table A8 in Appendix A with maximum average deviation of 0.31 mm and minimum of −0.04 mm.

### 3.6. Microstructure and Hardness of SLA-Based IC parts

**Figure 15:**
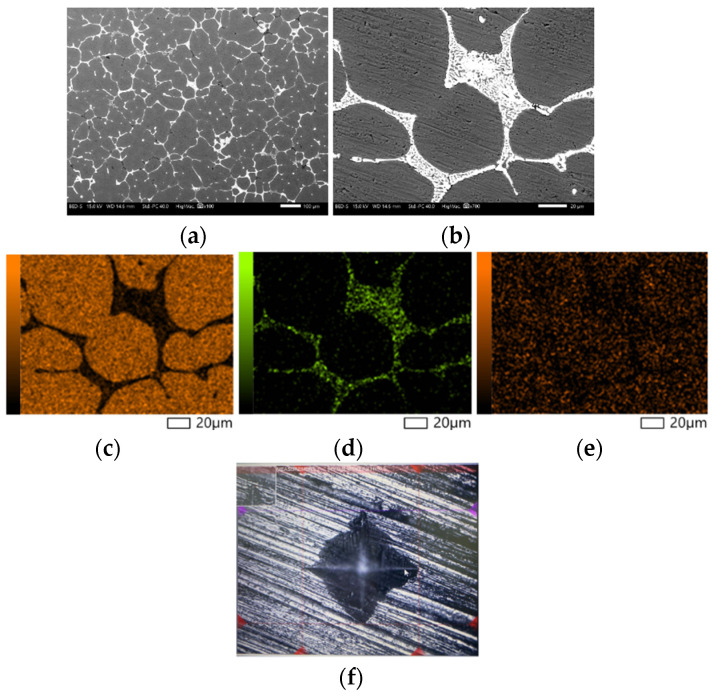
SEM of the SLA-based IC parts (**a**) Microstructure (×100); (**b**) Microstructure (×700); (**c**) Al; (**d**) Cu; (**e**) Mg; (**f**) Microhardness (HV) tester interface.

The SLA-based IC parts were characterized using the SEM, Vickers hardness test, and microstructure analysis. They had a microhardness of 101.6 HV with a standard deviation of 0.7. According to the energy dispersive spectroscopy (EDS) results, Aluminum, Copper, and Magnesium are present in the SLA-based IC samples with percentages by weight of 94.3 %, 4.25%, and 1.45%, respectively. The interface of Microhardness (HV) tester and microstructure of the SLA-based IC parts are shown in Figure 15.

## 4. Conclusions

This study examined the effect of process parameters on the surface roughness and dimensional accuracy of the SLA patterns by using the small-scale prosthetic hip implant as a benchmark model. The Taguchi L18 array was used for DOE and fifty-four SLA patterns were printed for the measurements. The optimum parameters which reduce the surface roughness and dimensional deviation of the SLA printed parts and the SLA-based IC parts were established. The main findings of the study are as follows:

The most influential SLA printing parameter affecting the surface roughness was the build angle, followed by the layer thickness. The optimum values that minimize the surface roughness are 0.025 mm layer thickness, 0° build angle, 1.0 support density, and across the front base orientation. As for the dimensional accuracy, the optimum printing parameters are a 0.025 mm layer thickness, 30° build angle, 0.6 support density, and diagonal to the front base orientation.

The SLA-based IC components had the optimum values for the surface roughness such as 0.025 mm layer thickness, 0° build angle, 1.0 support density, and parallel to the front base orientation. As for the dimensional accuracy, the optimum printing parameters are 0.050 mm layer thickness, 45° build angle, 0.8 support density, and diagonal to the front base orientation.

## Figures and Tables

**Table 1:** Summary of SLA studies.

Source	Printing Process Parameters	Properties	Materials	Findings about Parameters Optimization
[11]	Layer thickness	Surface roughness, dimensional stability	V3 (FLGPR03) grey resin	- No effect of layer thickness on surface roughness - A 25 µm layer thickness for dimensional accuracy
[12]	Orientation in relation to the printer’s build platform	Dimensional accuracy, surface roughness	Denture base polymethyl methacrylate (PMMA)	- A 90 degree angle resulted in the lowest dimensional errors, and surface roughness
[13]	Layer thickness, model structure, model base orientation, position build platform	Surface roughness	V3 (FLGPR03) grey resin	- No effect of model structure and build platform position was found
[14]	Build orientation (front, side, top), angular orientation around all the three-principal axis	Surface roughness	Visijet Sl Clear	- Surface roughness is not statistically significantly affected by altering the build orientation, However, it is influenced by changing the axis or angle - A 90° orientation along the *x* and *z*-axis resulted in lowest roughness
[15]	Printing orientation Position on build platform Layer thickness	Dimensional accuracy	Formlabs Clear Resin	Dimensional accuracy was improved when printing with - lower layer thickness (0.025 mm) - in center of the platform
[16]	Build orientation, positioning	Dimensional accuracy	Dental SG resin	- Used 45° orientation - Positioning in the center of the platform were recommended to be used
[17,18]	Build angle in relation to the printer’s build platform	Dimensional accuracy	Dental SG Resin	- The 0 and 45-degree build angles resulted in surgical templates with the highest level of precision
[17]	Build angle in relation to the *z*-axis	Accuracy (trueness and precision)	Formlabs Clear Resin	- Printing at a 45-degree angle yielded superior accuracy when compared to printing at 0 or 90 degrees
[19]	Layer thickness	Dimensional accuracy	Grey and Cast resin	- No statistically significant variations in accuracy between the three different layer thicknesses
[20]	Build angle with respect to the build platform	Dimensional accuracy	Dental LT Clear Resin	- The general accuracy is not greatly affected by the print orientation
[21]	Layer thickness (0.025 and 0.050 mm) Part orientation (horizontal, slanted, vertical in relation to the build platform)	Dimensional accuracy, surface roughness	Formlabs Rigid 10K V0 and High Temperature V02	- The 0.050 mm layer thickness led to low shape quality - Surface roughness was minimized due to the horizontal orientation

**Table 2:** Resin characteristics [28].

Casting Properties		Mechanical Properties	
Wax content	20%	Young’s Modulus	220 MPa
Ash content	0.0–0.1%	Tensile Strength	12 MPa
		Elongation at break	13%

**Table 3:** SLA printing parameters and control levels.

Parameters	Level 1	Level 2	Level 3
A: Layer thickness (mm)	0.025	0.050	-
B: Build angle (°)	0	30	45
C: Support density	0.6	0.8	1
D: Model base orientation	Parallel to the front	Across the front	Diagonal to the front

**Table 4:** Design of experiments (DOE) for SLA prints.

No.	A	B	C	D
1	1	1	1	1
2	1	1	2	2
3	1	1	3	3
4	1	2	1	1
5	1	2	2	2
6	1	2	3	3
7	1	3	1	2
8	1	3	2	3
9	1	3	3	1
10	2	1	1	3
11	2	1	2	1
12	2	1	3	2
13	2	2	1	2
14	2	2	2	3
15	2	2	3	1
16	2	3	1	3
17	2	3	2	1
18	2	3	3	2

**Table 5:** SLA printing parameters and control levels.

Dimensions	Radius	Width	Length	Thickness
Features	Head	Neck	Stem	Stem
			Neck	Neck

**Table 6:** Dimensional deviation of the SLA printed parts.

Dimension	Features	Nominal Value (mm)	Average Measured Value (mm)	Range		Average Deviation (mm)
				Max (mm)	Min (mm)	
Radius	Sphere	7.00	7.12	7.50	6.56	0.12
Width	Neck	6.00	5.81	5.92	5.64	−0.19
Thickness	Stem	3.00	3.10	3.54	2.82	0.10
	Neck	3.00	3.09	3.58	2.78	0.09
Length	Stem	48.00	47.79	48.92	47.50	−0.21
	Neck	7.50	7.57	7.89	7.38	0.07

**Table 7:** Results of surface roughness (SLA printed patterns).

Experiment Run No.	Surface Roughness Ra (µm)	Standard Deviation (µm)	S/N Ratio (dB)
1	0.922	0.060	0.702
2	0.882	0.052	1.091
3	1.023	0.118	−0.199
4	1.210	0.068	−1.655
5	1.398	0.140	−2.908
6	1.367	0.111	−2.717
7	1.466	0.235	−3.325
8	1.619	0.179	−4.183
9	2.355	0.799	−7.439
10	1.205	0.226	−1.621
11	0.987	0.095	0.115
12	1.111	0.087	−0.917
13	2.860	0.102	−9.127
14	2.927	0.306	−9.328
15	2.629	0.099	−8.395
16	2.875	0.624	−9.172
17	2.861	0.536	−9.131
18	2.028	0.662	−6.141

**Table 8:** Response table for the means for surface roughness of SLA printed parts.

Level	Layer Thickness (mm)	Build Angle (°)	Support Density	Model Base Orientation
1	1.360	1.022	1.756	1.827
2	2.165	2.065	1.779	1.624
3	-	2.201	1.752	1.836
Delta	0.804	1.179	0.027	0.212
Rank	2	1	4	3

**Table 9:** ANOVA table for surface roughness of SLA parts.

Source	DOF	Seq SS	Adj SS	Adj MS	F	P	Contribution %
Layer thickness (mm)	1	60.803	60.803	60.803	16.6179	0.00223	24.66
Build angle (°)	2	145.755	145.755	72.877	19.9181	0.00033	59.11
Support density	2	264	264	0.132	0.0361	0.96468	0.10
Model base Orientation	2	3.155	3.155	1.578	0.4312	0.66128	1.28
Residual error	10	36.589	36.589	3.659			14.84
Total	17	246.565					100

**Table 10:** Dimensional deviation of the SLA-based IC parts.

Dimension	Features	Nominal Value (mm)	Average Measured Value (mm)	Range		Average Deviation (mm)
				Max (mm)	Min (mm)	
Radius	Sphere	7.00	7.15	7.51	6.89	0.15
Width	Neck	6.00	5.92	6.08	5.74	−0.08
Thickness	Stem	3.00	3.12	3.75	2.68	0.12
	Neck	3.00	3.10	3.58	2.51	0.10
Length	Stem	48.00	47.60	47.93	47.16	−0.40
	Neck	7.50	7.48	7.56	7.37	−0.02

**Table 11:** Results of surface roughness (SLA-based IC patterns).

Experiment Run No.	Surface Roughness Ra (µm)	Standard Deviation (µm)	S/N Ratio (dB)
1	1.406	0.072	−2.961
2	1.650	0.047	−4.349
3	1.504	0.522	−3.542
4	1.766	0.047	−4.939
5	2.879	0.047	−9.185
6	2.465	0.264	−7.836
7	3.139	0.737	−9.935
8	3.038	0.998	−9.653
9	2.805	0.696	−8.959
10	3.003	0.047	−9.552
11	2.330	0.516	−7.349
12	1.576	0.118	−3.951
13	2.667	0.687	−8.522
14	2.527	0.610	−8.053
15	2.658	0.674	−8.491
16	2.798	0.047	−8.936
17	2.440	0.562	−7.759
18	2.983	0.861	−9.492

**Table 12:** Response table for means for surface roughness of SLA-based IC components.

Level	Layer Thickness (mm)	Build Angle (°)	Support Density	Model Base Orientation
1	2.305	1.912	2.463	2.265
2	2.564	2.494	2.478	2.482
3	-	2.868	2.332	2.547
Delta	0.259	0.956	0.146	0.282
Rank	2	1	4	3

**Table 13:** ANOVA table for surface roughness of the SLA-based IC parts.

Source	DOF	Seq SS	Adj SS	Adj MS	F	P	Contribution %
Layer thickness	1	6.414	6.414	6.414	1.81	0.208	6.86
Build angle	2	45.804	45.804	22.902	6.46	0.016	48.98
Support density	2	1.416	1.416	0.708	0.20	0.822	1.51
Model base orientation	2	4.438	4.438	2.219	0.63	0.554	4.75
Residual error	10	35.445	35.445	3.545			37.90
Total	17	93.517					100

## Data Availability

Data are contained within the article.

## Appendix A

**Table A1:** Mean ranking of parameters for dimensional accuracy of SLA parts.

Feature	Layer Thickness (mm)	Build Angle (°)	Support Density	Model Base Orientation
Neck width	3	2	4	1
Neck length	3	4	1	2
Neck thickness	4	1	3	2
Stem length	2	1	3	4
Stem thickness	4	1	2	3
Sphere radius	4	1	2	3
Sum	20	10	15	15
Ranking of sums	3	1	2	2

**Table A2:** Optimum printing parameters for dimensional accuracy of SLA parts.

Feature	Layer Thickness (mm)	Build Angle (°)	Support Density	Model Base Orientation
Neck width	0.025	30	1	Parallel to the front
Neck length	0.050	0	0.6	Across the front
Neck thickness	0.025	30	0.6	Diagonal to the front
Stem length	0.025	30	0.6	Across the front
Stem thickness	0.025	30	0.6	Diagonal to the front
Sphere radius	0.050	30	0.6	Diagonal to the front
Optimal level	0.025	30	0.6	Diagonal to the front

**Table A3:** Optimized printing parameters for dimensional accuracy and surface roughness of SLA parts.

Parameters	Dimensional Accuracy (mm)		Surface Roughness (µm)	
	Level	Value	Level	Value
A: Layer thickness (mm)	1	0.025	1	0.025
B: Build angle (°)	2	30	2	30
C: Support density	1	0.6	1	0.6
D: Model base orientation	3	Diagonal to the front	3	Diagonal to the front

**Table A4:** Dimensional deviation of the SLA parts printed with optimized parameters.

Dimension	Features	Nominal Value (mm)	Average Measured Value (mm)	Range		Average Deviation (mm)
				Max (mm)	Min (mm)	
Radius	Sphere	7.00	7.02	7.04	7.00	0.02
Width	Neck	6.00	5.85	5.89	5.83	−0.15
Thickness	Stem	3.00	2.99	3.00	2.98	−0.01
	Neck	3.00	2.99	3.05	2.94	−0.01
Length	Stem	48.00	48.03	48.06	48.01	0.03
	Neck	7.50	7.56	7.57	7.52	0.06

**Table A5:** Mean ranking of parameters for dimensional accuracy of SLA-based IC parts.

Feature	Layer Thickness (mm)	Build Angle (°)	Support Density	Model Base Orientation
Neck width	4	1	2	3
Neck length	1	2	4	3
Neck thickness	2	1	3	4
Stem length	1	2	3	4
Stem thickness	4	1	3	2
Sphere radius	4	1	3	2
Sum	16	8	18	18
Ranking of sums	2	1	3	3

**Table A6:** Optimum printing parameters for dimensional accuracy of SLA-based IC parts.

Feature	Layer Thickness (mm)	Build Angle (°)	Support Density	Model Base Orientation
Neck width	0.025	30	0.8	Across the front
Neck length	0.025	30	0.8	Diagonal to the front
Neck thickness	0.050	45	1.0	Diagonal to the front
Stem length	0.050	0	0.8	Across the front
Stem thickness	0.050	45	1.0	Diagonal to the front
Sphere radius	0.050	45	0.6	Diagonal to the front
Optimal level	0.050	45	0.8	Diagonal to the front

**Table A7:** Optimized printing parameters for dimensional accuracy and surface roughness of SLA-based IC components.

Parameters	Dimensional Accuracy (mm)		Surface Roughness (µm)	
	Level	Value	Level	Value
A: Layer thickness (mm)	2	0.050	1	0.025
B: Build angle (°)	3	45	1	0
C: Support density	2	0.8	3	1
D: Model base orientation	3	Diagonal to the front	1	Parallel to the front

**Table A8:** Dimensional deviation of the optimized SLA-based IC parts.

Dimension	Features	Nominal Value (mm)	Average Measured Value (mm)	Range		Average Deviation (mm)
				Max (mm)	Min (mm)	
Radius	Sphere	7.00	7.05	7.13	6.94	0.05
Width	Neck	6.00	5.88	6.09	5.73	−0.12
Thickness	Stem	3.00	3.06	3.17	2.84	0.06
	Neck	3.00	3.04	3.20	2.91	0.04
Length	Stem	48.00	47.69	47.93	47.16	−0.31
	Neck	7.50	7.35	7.46	7.24	−0.15
